# Media Use Among Students From Different Health Curricula: Survey Study

**DOI:** 10.2196/12809

**Published:** 2019-08-19

**Authors:** Michaela Zupanic, Patrick Rebacz, Jan P Ehlers

**Affiliations:** 1 Personality Psychology and Diagnostics Department of Psychology, Faculty of Health Witten/Herdecke University Witten Germany; 2 Didactics and Educational Research in Healthcare Department of Medicine, Faculty of Health Witten/Herdecke University Witten Germany

**Keywords:** social media, medical education, computers, interprofessional relations, distance education, health occupations

## Abstract

**Background:**

Mobile devices such as smartphones, tablets, and laptop computers enable users to search for information and communicate with others at any place and any time. Such devices are increasingly being used at universities for teaching and learning. The use of mobile devices by students depends, among others, on the individual media literacy level and the curricular framework.

**Objective:**

The objective of this study was to explore whether there were differences in media use in students from various curricula at the Faculty of Health, Witten/Herdecke University.

**Methods:**

During the 2015-16 winter term, a survey was conducted at the Faculty of Health, Witten/Herdecke University, in which a total of 705 students (out of 1091 students; response rate: 705/1091, 64.61%) from 4 schools participated voluntarily: medicine (346/598), dentistry (171/204), psychology (142/243), and nursing science (46/46). The questionnaire developed for the study included 132 questions on 4 topics: (1) electronic and mobile devices (19 questions), (2) communication and organization of learning (45 questions), (3) apps/programs/websites/media (34 questions), and (4) media literacy (34 questions). The questionnaire was distributed and anonymously completed during in-class courses.

**Results:**

Students from all 4 schools had at least two electronic devices, with smartphones (97.4%, 687/705) and laptops (94.8%, 669/705) being the most common ones. Students agreed that electronic devices enabled them to effectively structure the learning process (mean 3.16, SD 0.62) and shared the opinion that university teaching should include imparting media literacy (mean 2.84, SD 0.84). Electronic device ownership was the highest among medical students (mean 2.68, SD 0.86) and medical students were the only ones to use a tutorial (36.1%, 125/346). Dental students most widely used text messages (mean 3.41, SD 0.49) and social media (mean 2.57, SD 1.10) to organize learning. Psychology students considered mobile devices to be most ineffective (mean 2.81, SD 0.83). Nursing science students used emails (mean 3.47, SD 0.73) and desktop computers (39%, 18/46) most widely.

**Conclusions:**

The results show that almost all students use electronic learning (e-learning) tools. At the same time, different profiles for different degree programs become apparent, which are to be attributed to not only the varying curricula and courses but also to the life circumstances of different age groups. Universities should, therefore, pay attention to the diverse user patterns and media literacy levels of students when planning courses to enable successful use of e-learning methods.

## Introduction

### Background

The ubiquitous distribution of mobile devices and internet access support mobile learning as a new and global trend in education [1,2]. The use of mobile devices such as smartphones, tablets, and laptop computers for obtaining information and communicating course content enables users to learn at any place and time, in different contexts, various situations, and by interacting with others [3-5]. Mobile learning occurs when the learner is not at a permanent and fixed location or if he/she uses mobile technologies for learning [6]. In this context, mobility comprises 3 aspects: technology mobility, learners’ mobility, and learning process mobility [7] and is thus understood as a part of electronic learning (e-learning).

By means of electronically arranged digital learning tools, course content is presented as multimedia content and thus supports interactive and self-directed learning [8,9]. This may take place within given instruction structures or in network structures for self-directed learning, such as virtual learning spaces and blended learning. Virtual learning spaces facilitate a physical separation between teachers and learners by means of the internet as a communication medium. Traditional lectures are combined with the advantages of e-learning [10]. Blended learning merges Web-based phases with in-class teaching and makes use of networking opportunities via the internet using conventional learning methods [11,12]. Modern learning environments and apps even allow the mobile use of virtual reality and augmented reality in medical learning [13].

The use of mobile devices in teaching and learning, however, also gives rise to controversy as, in addition to its advantages, it may also involve disruptive components, such as distraction, mingling of private and professional matters, inadequate technologies, students owning different equipment, or cognitive overload of users [14-16]. Some lecturers recoiled from the effort and the creation of learning apps or believed that computers and the internet were sources of distraction, which disrupted teaching and learning and thus impaired the understanding of the subject matter [17-19]. Most commonly, however, the reason for problems in technology-enhanced learning is inadequate didactics [20]. Educational challenges to be met are the adaptation of instruction type and content, as well as a joint creation of learning tools by teachers and students according to the students’ learning levels [21,22]. Sustainable embedding of digital learning elements in higher education teaching, therefore, requires also the development of teachers’ digital literacy, as well as an adapted and innovative culture of teaching and learning [23-25].

### Objective

Given the fact that Witten/Herdecke University (UW/H) strives to break new educational ground and provide impetus for research-based development of teaching and learning [26], this study aims to cast a glance at the status quo regarding the equipment and digital media learning at the Faculty of Health. While being the only university in the German-speaking region with a Faculty of Health comprising the 4 schools of medicine, dentistry, psychology, and nursing science, UW/H places special emphasis on interprofessional education when designing the curricula of the individual degree programs. To facilitate digitally supported interprofessional education, students were surveyed to identify similarities and differences between the individual disciplines with respect to media use and develop profiles of the respective schools and curricula.

## Methods

The Ethics Committee of UW/H voted in favor of the concept of this study (application number: 144/2015).

### Study Design and Participants

The cross-sectional study was conducted at UW/H at the beginning of the 2015-16 winter term at the Faculty of Health, which comprises the 4 schools of medicine, dentistry, psychology, and nursing science. The questionnaires were distributed to the students in the schools at different times, with the aim of generating the greatest possible response rate: medicine and psychology during the compulsory progress test and dentistry and nursing science during course-specific compulsory courses. Students completed the questionnaire voluntarily and anonymously during in-class courses. No other personal data other than the degree program, gender, and age group were collected to avoid reidentifiability of individuals, which would otherwise have been possible because of the small cohorts per semester (medicine: 42 students, dentistry: 44 students, psychology: 35 students, and nursing science: 15 students) and the family study situation at the UW/H with learning in small groups.

Of the 1091 students of the Faculty of Health, 705 students completed the questionnaire (medicine: 346/598, dentistry: 171/204, psychology: 142/243, and nursing science: 46/46; total response rate of 65% students); 20 incomplete questionnaires were excluded. There were significant differences regarding the gender ratio (χ^2^_3_=30.4; *P*<.001) between the schools. Most students at the school of nursing science were women (>90%, 42/46), whereas the schools of medicine and dentistry had the largest share of men, with more than 40% each (137/346 and 72/171, respectively). Schools also differed significantly regarding the age groups (χ^2^_12_=438.1; *P*<.001). Although more than half of the medical, dental, and psychology students interviewed were aged between 21 and 25 years, more than 90% (43/46) of the nursing science students were aged >26 years. More than 90.8% (129/142) of the psychology students were aged <25 years.

### Questionnaire Development

A questionnaire was developed to answer the research questions. The questionnaire was compiled in a multiple-sample process based on the literature, brainstorming sessions, and discussions, as well as results and experiences from a pilot study of mobile learning at UW/H in the 2015 summer term. Semistructured, personal-expert interviews were conducted until saturation of categories was reached with 10 psychology and 8 medical students. Results of content analysis made it clear that e-learning media were used (ie, computers, smartphones, and apps). On the basis of these results in this study, the research question is being extended to learning with digital media. The developed questionnaire included open and closed questions (4-point Likert scale from *1=no, not at all* to *4=yes, absolutely*) on the following topics: (1) electronic and mobile devices, with 19 questions on the possession of devices and their use in everyday life, (2) communication and the way learning is organized, with 45 questions on the search for information and the organization of learning, (3) apps/programs/websites/media, with 34 questions on the apps used, and (4) media literacy, with 34 questions on students’ assessment of whether they consider mobile learning as impeding or useful.

The focus of this study was on the topics (2) communication and learning organization and (4) media literacy, here, especially the evaluation of UW/H duties. Both scales show a very good internal consistency with Cronbach alpha of .926 for communication and learning organization and .869 for media literacy.

### Statistical Analysis

The data were analyzed first by descriptive statistics (means and standard deviations) using the SPSS software package (Statistical Package for the Social Sciences, version 24 for Windows, IBM Corporation). Nonparametric group comparisons between the schools were carried out using the chi-square test in case of categorical response formats and the Kruskal-Wallis test for several independent groups in case of 4-point scale formats. Results were considered significant with an error probability of 5% (*P*<.05). Effect sizes (Cohen *d*) were calculated on the Web [27] and interpreted as small (0.20), medium (0.50), or large (0.80) [28].

## Results

### Similarities Between the Schools of the Faculty of Health

**Figure 1:**
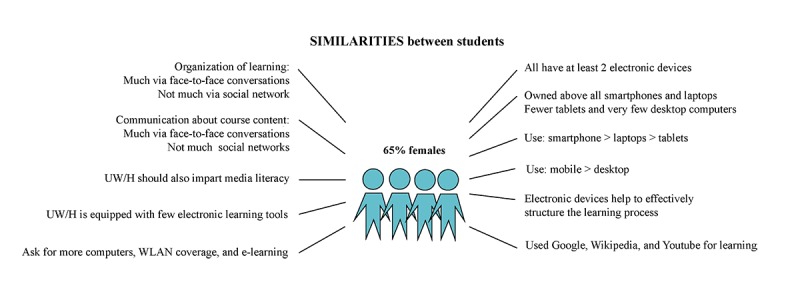
Similarities between students from all 4 schools of the Faculty of Health regarding their media use (n=705). UW/H: Witten/Herdecke University; WLAN: wireless local area network.

Students from the various schools showed some similarities, as shown in Figure 1. Students of all 4 schools owned at least 2 electronic devices on average; smartphones (97.4%, 687/705) and laptop computers (94.8%, 669/705) in particular were most common. Fewer students had tablet computers (45.9%, 324/705), and very few still owned desktop computers (16.0%, 113/705). All agreed that electronic devices help to effectively structure the learning process (mean 3.16, SD 0.62; range *1=no, not at* all to *4=yes, absolutely*). Students used mobile devices to look up and search for information (mean 3.18, SD 0.68), as well as Google, Wikipedia, YouTube, PubMed, and DocCheck. Mobile devices were less frequently used for organizing the learning process (mean 2.63, SD 0.94) and communicating about the course content (mean 2.54, SD 0.88). The same applies to social networks regarding organization (mean 2.17, SD 1.14) and communication (mean 1.95, SD 1.07). Face-to-face conversations were preferred most by the students for organization (mean 3.39, SD 0.82) and communication (mean 3.81, SD 0.48). Students from all schools shared the opinion that teaching at UW/H should also comprise imparting media literacy (mean 2.84, SD 0.84). However, there was, above all, a shortage of computers (n=58 of a total of 171 mentions), wireless local area network coverage (n=28), and e-learning courses (n=103).

### Differences Between the Schools of the Faculty of Health

**Figure 2:**
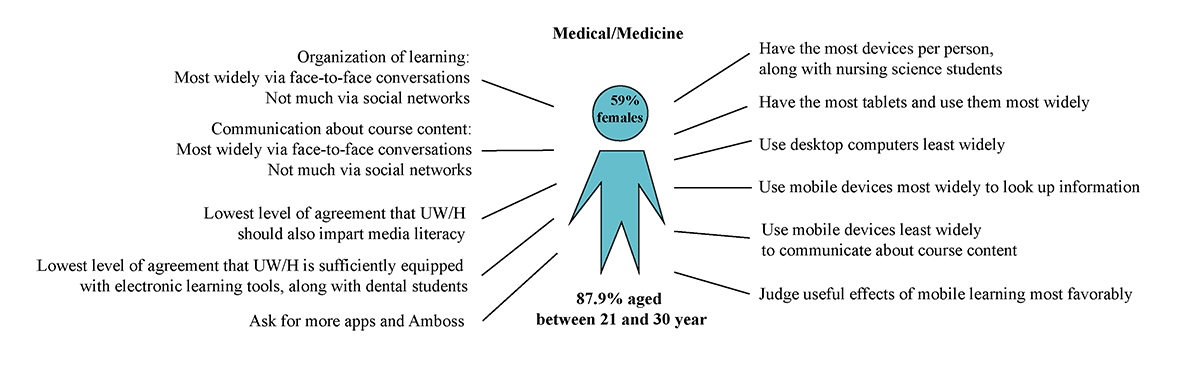
Media use characteristics of students from the School of Medicine, Faculty of Health (n=346). UW/H: Witten/Herdecke University.

**Figure 3:**
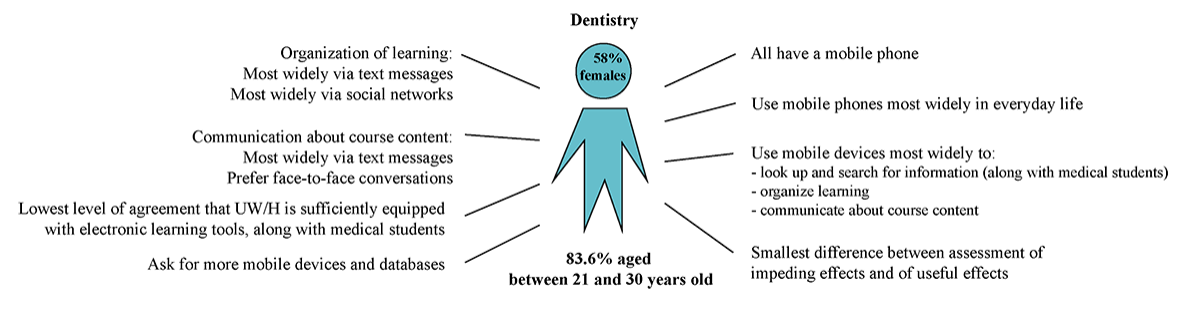
Media use characteristics of students from the School of Dentistry, Faculty of Health (n=171). UW/H: Witten/Herdecke University.

**Figure 4:**
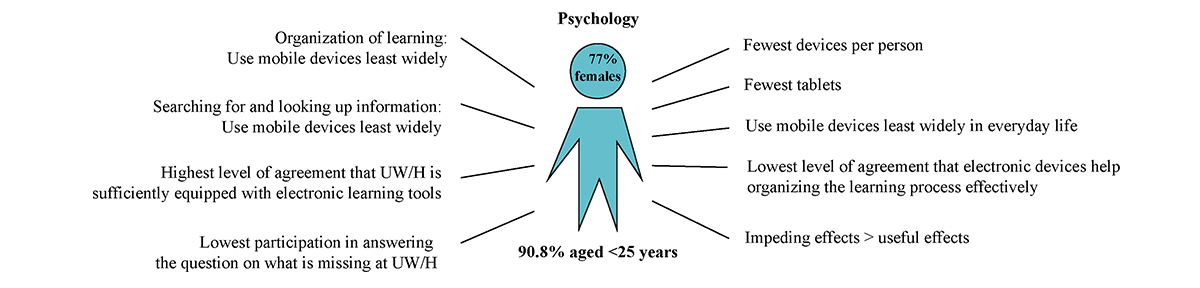
Media use characteristics of students from the School of Psychology and Psychotherapy, Faculty of Health (n=142). UW/H: Witten/Herdecke University.

**Figure 5:**
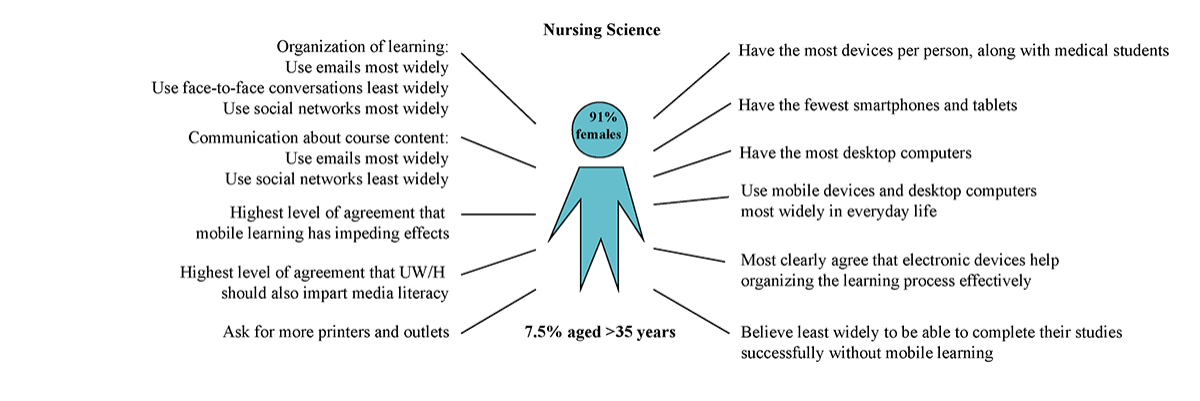
Media use characteristics of students from the School of Nursing Science, Faculty of Health (n=46). UW/H: Witten/Herdecke University.

Despite these similarities, however, there were also significant differences between students from different schools. These differences are elaborated in the form of profiles and demonstrated in Figures 2-5. *Medical students* owned the most electronic devices (mean 2.68, SD 0.86; range *1=no, not at all* to *4=yes, absolutely*), had the most tablet computers (52.8%, 183/346), and used them most frequently (mean 2.55, SD 1.24). Medical students were the only ones to use a uniform learning program called Amboss (36.1%, 125/346). They most clearly felt that mobile learning contributes to successful learning (mean 3.14, SD 0.77). Together with the dental students, they least agreed that UW/H is sufficiently equipped with electronic devices (mean 2.06, SD 0.81). Medical students participated the most in answering the question on what is missing at UW/H (88/346, 25.4%) and mentioned, above all, apps and access, in particular to the Amboss learning software (23/346, 6.6%), in addition to computers and e-learning.

All *dental students* had at least one smartphone and used it most often (mean 3.94, SD 0.25). They used mobile devices most frequently to search for and look up information (mean 3.27, SD 0.81), organize learning (mean 2.96, SD 1.12), and communicate about course content (mean 2.77, SD 0.84). Dental students were the only ones to mention the UW/H learning platform Moodle. Compared with the other schools, they used text messages (mean 3.41, SD 0.49) and social networks (mean 2.57, SD 1.10) most widely to organize learning. Along with psychology students, they showed the highest level of agreement that mobile devices distracted from learning (mean 2.77, SD 0.94). They asked for more databases (11/171, 6.4%) and mobile devices (8/171, 4.6%).

*Psychology students* had the least devices (mean 2.42, SD 0.75) and used them least widely in everyday life (mean 3.20, SD 0.56). They had the lowest share of tablets (23.2%, 33/142) and showed the lowest level of agreement about using mobile devices to search for and look up information (mean 2.89, SD 0.63) and organize learning (mean 2.43, SD 0.74). Very few thought that electronic devices support the learning process (mean 2.81, SD 0.83). Along with nursing science students, psychology students rated the contribution of mobile learning to successful learning as the lowest (mean 2.71, SD 0.72). They showed the highest level of agreement that UW/H is sufficiently equipped with electronic devices (mean 2.57, SD 0.80).

*Nursing science students* owned, along with medical students, the most devices per person (mean 2.67, SD 1.01). They had the most desktop computers (39%, 18/46) and used them most widely in everyday life (mean 2.06, SD 1.29). They showed the highest level of agreement that electronic devices support the learning process (mean 3.17, SD 0.95), and most of them shared the opinion that UW/H should also impart media literacy (mean 3.33, SD 0.69). Nursing science students used emails most widely for organization purposes (mean 3.47, SD 0.73) and communication about course content (mean 3.18, SD 0.96). They used social networks least widely for organization (mean 1.32, SD 0.84) and communication purposes (mean 1.19, SD 0.70).

### Communication and Learning Organization Such as Media Literacy

A direct comparison of the 4 schools showed some differences in the 2 considering scales of the questionnaire (see Table 1). The 4 categories of the scale Communication and Learning (CL), based on 3 items each, are as follows: CL learning, for example, *“* Electronic devices help me to make my learning process more effective.”; CL organization, for example, “I use the devices to organize my studies.”; CL communication, for example, *“* I use the devices to communicate and discuss learning content with others *.* ”; and CL information, for example, “I use devices to look up and search for information for my studies.” The difference with the largest effect size is clear for CL learning. For CL organization and CL communication there are only weak effect sizes, and the results for CL information do not differ between the schools. There are 2 categories of the scale Media Literacy (ML): ML positive with 8 items, for example, “I think electronic learning on mobile devices foster the learning success.” and ML negative with 10 items, for example, “Electronic learning on mobile devices is rather inhibitive.” Both categories of the scale ML show slight differences between the schools with weak effect sizes. Comparisons concerning the UW/H equipment (“The UW/H is sufficiently equipped with electronic learning media.”), media literacy (“The University teaching at the UW/H should, therefore, provide the mediation of media literacy.”), and lecturers (“I was helped handling mobile devices by my lecturers.”) resulted in slight differences with weak effect sizes, too.

**Table 1:** Differences between students from all 4 schools of the Faculty of Health regarding their media use.

Scales	Medicine, mean (SD)	Dentistry, mean (SD)	Psychology, mean (SD)	Nursing science, mean (SD)	Chi-square value (*df*)	*P* value	Cohen *d*
CL^a^ learning	1.96 (1.04)	1.40 (0.72)	1.19 (0.53)	1.33 (0.73)	88.4 (3)	<.001	0.747
CL information	3.16 (0.60)	3.11 (0.59)	2.88 (0.48)	2.86 (0.69)	4 (3)	.26	0.076
CL organization	2.49 (0.78)	2.61 (0.69)	2.46 (0.57)	2.51 (0.69)	30.3 (3)	<.001	0.403
CL communication	2.20 (0.87)	2.53 (0.70)	2.39 (0.59)	2.37 (0.83)	21.6 (3)	<.001	0.330
ML^b^ positive	2.50 (0.51)	2.46 (0.47)	2.24 (0.68)	2.23 (0.60)	25.9 (3)	<.001	0.367
ML negative	2.73 (0.57)	2.54 (0.58)	2.50 (0.60)	2.74 (0.70)	20.6 (3)	<.001	0.321
UW/H^c^ equipment	2.06 (0.81)	2.04 (0.70)	2.57 (0.80)	2.15 (0.73)	31.3 (3)	<.001	0.410
UW/H media literacy	2.74 (0.85)	2.99 (0.79)	2.75 (0.84)	3.33 (0.69)	26.1 (3)	<.001	0.369
UW/H lectures	1.33 (0.64)	1.27 (0.56)	1.43 (0.76)	1.83 (1.12)	10.4 (3)	.02	0.206

^a^CL: Communication and Learning.

^b^ML: Media Literacy.

^c^UW/H: Witten/Herdecke University.

## Discussion

### Summary

The objective of the study was to identify differences in media use between different curricula at the Faculty of Health, UW/H. Our findings describe profiles that show that the students of medicine, dentistry, psychology, and nursing science clearly differ in media literacy and user behavior. All had in common that media literacy was not, or rarely, taught by the UW/H lectures. For successful use of electronic devices, the faculty has to take into account that participants of interprofessional groups may differ in media affinity and media literacy when implementing mobile learning. This may be considered disadvantageous with respect to initial training and coordination, regarding peer-to-peer learning; however, it can also be seen as an advantage, if this challenge of developing professionalism in compliance with one’s own Web privacy and that of patients is accepted by the students and faculty together. Therefore, the following guiding principle has been defined: *Know your students, use their skills, and guide their way* [29].

### Principal Findings

Medical students generally have a wide range of mobile devices [30]. Among the students of the Faculty of Health of UW/H, mobile devices were more common than at other universities, for example, at the Faculty of Medicine of the University of Münster [31] or students of Polytechnic State University of California [15]. This may be explained, on the one hand, by the rapid progress of media use and, on the other, by differences in income. For young people with a formal higher background, the internet is a much more important research and information medium that they use more frequently and more extensively for information search than people with low socioeconomic status [32]. UW/H is a private university and thus charges tuition fees that are rather high compared with other German universities, so that a relatively high socioeconomic status of the students can be assumed.

Many similarities between students became apparent. All mobile devices have been used in everyday life and for learning purposes, especially for looking up and quickly searching for information because clear information is easier to communicate through digital media [33]. To an extent comparable with that of the students at the University of Münster [31] and the University of California [30] and adolescents of the JIM study [32], UW/H students primarily used Google as an internet search engine to obtain information. YouTube played an important and Wikipedia played an even more increasingly important role in learning [34]. When students get the opportunity to learn how to use the technology, mobile devices and apps can be conducive to learning and improve learning [35]. However, lecturers should also be supported in the development of media literacy, so that they have the digital skills and abilities required to provide appropriate learning materials and tailor their courses to them [5,36,37]. The imparting of media literacy by the lecturers and the faculty is an important wish of the UW/H students, too.

There was less agreement about the use of mobile devices for organizational purposes in learning contexts. For these purposes, students most often preferred face-to-face conversations, despite the fact that a central digital program is available for organizing their studies [38]. However, the small number of students admitted per term, the course format (small group teaching and problem-based learning), and the favorable student-teacher ratio encourage students to organize learning via face-to-face conversations. Lessons and content that require interpretation and discussion, and that may also be ambiguous, cannot be communicated as effectively through mobile media as through traditional face-to-face contact [33]. When this study was conducted during the 2015-16 winter term, the UW/H Faculty of Health comprised 38 lecturers, 150 research assistants, and 341 contract lecturers for 1091 students [39].

There were significant differences with small effect sizes between students from the schools of medicine, dentistry, psychology, and nursing science of the Faculty of Health, which have to be discussed in the context of age and study conditions.

Medical students most frequently use mobile devices to search for and look up information and believe that mobile learning is crucial to their learning success. More than one-third of students already use the Amboss learning program, and one-quarter would like UW/H to provide the program. Dental students in particular used mobile learning most frequently to communicate about course content via text messages and organize learning. According to Walsh [25], students are increasingly expecting that all e-learning services will work well on mobile devices. The frequent use and handling of the mobile devices and programs, thereby, creates an awareness of their advantages and disadvantages and trains the use of technology [35]. Accordingly, the best-equipped students of medicine in this study wished the least for impartment of media literacy.

Psychology students were the youngest students within the cohort and used mobile devices least often in their everyday life and for learning. Nursing science students pursue a degree program for working professionals; hence, they are older than students from the other 3 schools and are, therefore, no digital natives [40,41]. Both studies have in common the large proportion of women with a known gender effect on the use of mobile devices [37]. In addition, it is generally assumed that today’s students, because of their young age in information technology (IT), have much more experience and are better educated than former students and faculty members aged >40 years [37]. However, this is a fallacy, as today’s students recognize their need for advanced IT skills and want to learn the skills needed for the digital age [42], as shown in this sample.

### Limitations

On the basis of a response rate of 64.61% (705/1091) with a gender ratio comparable with the total student sample, the authors assume that the findings are representative for the Faculty of Health, UW/H. Unfortunately, no additional information on the student semester could be given to look at any differences between undergraduate and postgraduate students. In combination with the person variables age and gender, it would, otherwise, be possible to reidentify individuals, because of the small cohorts per semester. However, this study’s findings cannot be generalized easily as the cohort surveyed was very small, and the curricular offers correspond to those of a university with model curricula.

### Conclusions

Mobile learning is being applied at UW/H. Electronic devices, mobile devices in particular, are very popular among the Faculty of Health students and used for learning purposes. Since 2015, the recommendations for existing e-learning modules are collected and evaluated by lecturers and made available [43]. Thus, in addition to self-directed learning, confidence in the reliability of Web-based materials is promoted [44]. However, it has just turned into an integral part of studies, with the introduction of a new model curriculum. The conditions for expanding digital education at UW/H are favorable as, for example, 1 of the foci of the new medicine model curriculum (as of the 2018-19 winter term) is on digital medicine. In addition, the university offers the new master’s degree program Digital Transformation and Social Responsibility (Master of Arts). Regular public events on digitization (called *Digitaler Salon*) have taken place since 2016, as well as a cross-faculty course for imparting digital literacy [45]. Synchronous interaction with one another and face-to-face conversations are still the most important for learning, owing, on the one hand, to the student-teacher ratio and, on the other hand, to Humboldt educational concept being implemented at UW/H. In this respect, too, student digital helpers are increasingly being used for organization and implementation with the aid of a university’s most important resource: its students!

## Abbreviations

CL: Communication and Learning
e-learning: electronic learning
IT: information technology
ML: Media Literacy
UW/H: Witten/Herdecke University
